# Human temperatures for syndromic surveillance in the emergency department: data from the autumn wave of the 2009 swine flu (H1N1) pandemic and a seasonal influenza outbreak

**DOI:** 10.1186/s12873-016-0080-7

**Published:** 2016-03-09

**Authors:** Samantha F. Bordonaro, Daniel C. McGillicuddy, Francesco Pompei, Dmitriy Burmistrov, Charles Harding, Leon D. Sanchez

**Affiliations:** University Emergency Medical Services, Gates Vascular Institute, Buffalo, NY USA; Department of Emergency Medicine, Saint Joseph Mercy Hospital, Ann Arbor, MI USA; Department of Emergency Medicine, University of Michigan, Ann Arbor, MI USA; Exergen Corporation, Watertown, MA USA; Department of Physics, Harvard University, Cambridge, MA USA; Data Scientist, Seattle, WA USA; Department of Emergency Medicine, Beth Israel Deaconess Medical Center, One Deaconess Road, W-CC2, Boston, 02215 MA USA; Harvard Medical School, Boston, MA USA; Previous address: Emergency Department of Beth Israel Deaconess Medical Center, Boston, MA USA

**Keywords:** Outbreak, Public health surveillance, Syndromic surveillance, Influenza, Emergency department, Emergency medical service, Fever, Timeliness

## Abstract

**Background:**

The emergency department (ED) increasingly acts as a gateway to the evaluation and treatment of acute illnesses. Consequently, it has also become a key testing ground for systems that monitor and identify outbreaks of disease. Here, we describe a new technology that automatically collects body temperatures during triage. The technology was tested in an ED as an approach to monitoring diseases that cause fever, such as seasonal flu and some pandemics.

**Methods:**

Temporal artery thermometers that log temperature measurements were placed in a Boston ED and used for initial triage vital signs. Time-stamped measurements were collected from the thermometers to investigate the performance a real-time system would offer. The data were summarized in terms of rates of fever (temperatures ≥100.4 °F [≥38.0 °C]) and were qualitatively compared with regional disease surveillance programs in Massachusetts.

**Results:**

From September 2009 through August 2011, 71,865 body temperatures were collected and included in our analysis, 2073 (2.6 %) of which were fevers. The period of study included the autumn–winter wave of the 2009–2010 H1N1 (swine flu) pandemic, during which the weekly incidence of fever reached a maximum of 5.6 %, as well as the 2010–2011 seasonal flu outbreak, during which the maximum weekly incidence of fever was 6.6 %. The periods of peak fever rates corresponded with the periods of regionally elevated flu activity.

**Conclusions:**

Temperature measurements were monitored at triage in the ED over a period of 2 years. The resulting data showed promise as a potential surveillance tool for febrile disease that could complement current disease surveillance systems. Because temperature can easily be measured by non-experts, it might also be suitable for monitoring febrile disease activity in schools, workplaces, and transportation hubs, where many traditional syndromic indicators are impractical. However, the system’s validity and generalizability should be evaluated in additional years and settings.

**Electronic supplementary material:**

The online version of this article (doi:10.1186/s12873-016-0080-7) contains supplementary material, which is available to authorized users.

## Background

Infectious disease control and treatment are most feasible when outbreaks can be recognized and characterized early. The emergency department (ED) increasingly acts as a gateway to the evaluation and treatment of acute illnesses, ranging from seasonal influenza to novel disease outbreaks, and has therefore become a key site for syndromic surveillance. Because the ED has the potential to both detect and warn the community about possible outbreaks or hazards, studies of the ED have multiplied as local syndromic surveillance has become technologically achievable. For example, Hiller et al. [1] noted 24 individual systems of ED-based syndromic surveillance of influenza in a 2013 review, and additional studies have been published since [2, 3].

Yet, there are substantial obstacles to effective surveillance of disease outbreaks at the local level. Data must provide clear indications of disease while also being easy to collect and scale, attributes that are often mutually inconsistent. Influenza provides a good example of these challenges. Surveillance by the United States Centers for Disease Control and Prevention (CDC) focuses on especially clear indicators of influenza, such as virologic testing and outpatient visits for influenza-like illness (ILI) [4, 5]. Because these indicators are difficult to collect rapidly and at scale, CDC surveillance has been limited to delayed weekly reports, and concerted efforts to improve timeliness and local coverage have been discontinued [6]. In contrast, Google Flu Trends focused on search queries, which Google can easily acquire in real-time and with extensive local coverage [7]. However, flu search queries appear to be sensitive to factors that are misrepresentative of influenza, such as news stories, and extraneous events, such as changing search algorithms. As a result, Google Flu Trends repeatedly misestimated influenza [8–10], and the Google Flu program was recently closed down [11]. At the same time, Google also stopped publishing a similar website that was designed to monitor dengue fever (Google Dengue Trends [12]).

In this study, we present body temperature as a syndromic indicator that may offer a good balance of objectivity and ease of collection, and therefore might address some of the limitations to previous methods of local disease surveillance, both for influenza and for other diseases. As a clear indicator of fever, body temperature offers an objective means of surveillance for febrile diseases such as influenza, dengue, Severe Acute Respiratory Syndrome (SARS), and Ebola. Since temperature is routinely measured already, a system that allows it to be collected passively—without additional time or effort from the user—could be widely applicable. Further, body temperature is one of the few health measurements that is understood by laypersons. Consequently, it could also be applied for disease surveillance in non-clinical settings, such as schools and workplaces.

With these potential advantages in mind, we implemented a system of automated temperature collection and deployed this system in an active ED. This technical advance article presents the system’s implementation and the general features of the collected data, including comparisons with regional disease surveillance.

## Methods

### Ethics statement

This study was approved and consent was waived by the Beth Israel Deaconess Medical Center Institutional Review Board (protocol number: 2008-P-000412).

### Temperature monitoring

Temperature monitoring using the model TAT-5000 Exergen temporal artery thermometer (Exergen, Corp., Watertown, MA) was initiated in the triage area of an urban ED as a part of initial triage vital signs. Using this model, temperatures are measured by sliding the infrared thermometer across the forehead, a low-contact method that is expected to reduce the potential for disease transmission from the patient. Thermometers connected to data-logging modules replaced prior methods of measuring temperature at triage, such as oral and tympanic measurements. Included temperature data were collected between September 10, 2009 and August 29, 2011. Two to four data-logging thermometers were generally in use during this period (daily mean: 2.97, standard deviation [SD]: 1.08, range, 1–5), with exceptions for general maintenance. Thermometers were checked, time-stamped measurements were collected, and maintenance was performed on a roughly biweekly basis. Three thermometers were located at triage stations and one was located on a rolling unit. The data-logging thermometers were used as a surrogate to investigate the capabilities of real-time data reporting with networked wireless thermometers.

### Inclusion criteria and temperature preprocessing

The study population comprised persons presenting at an ED (Boston, MA) who underwent an initial triage vital signs assessment performed with a data-logging thermometer. All such persons were included, regardless of time of day, age, gender, or chief complaint. General characteristics of presentations at the ED are summarized in Table 1. Temperature collection was not linked to other hospital records, thereby providing a conservative assessment of the value of body temperature data alone.

**Table 1:** General characteristics of cases presenting at the emergency department, September 2009 through August 2011

Characteristic	Value
Visits	110,465
Sex	
Female	54 %
Male	46 %
Mean age	50.2 y
Rate of admission	37 %
Time of triage^a^	
10:00 PM–1:59 AM	11 %
2:00 AM–5:59 AM	6 %
6:00 AM–9:59 AM	11 %
10:00 AM–1:59 PM	26 %
2:00 PM–5:59 PM	25 %
6:00 PM–9:59 PM	21 %

^a^Assessed from the temperature measurement records

In traditional clinical studies, inclusion criteria are used to isolate the study from factors that could reduce precision or introduce bias. However, in automated surveillance, even the smallest requirement for additional human intervention in the data collection process represents a substantial barrier to scaling and practical implementation. Accordingly, the data-logging thermometers were designed to operate exactly as standard TAT-5000 models during measurement, without requiring any additional steps. Every temperature was recorded and considered to meet the inclusion criteria, including properly measured temperatures, improperly measured temperatures, repeated measurements of the same patient, and even accidental measurements, for example of the ED floor. Instead of coming from an idealized setting supplemented with extensive patient information, the temperatures are therefore representative of data that would actually be available under a real-time surveillance program.

During the study period, 89,856 temperature recordings were collected. Before our statistical analyses, we used two approaches to filter out temperature measurements that were unlikely to be individual body temperatures. First, temperature recordings below 95.0 °F (35.0 °C) were removed (11.6 %, *n* = 10,466) because they are predominantly mis-measurements. The rare human temperatures <95.0 °F (<35.0 °C) constitute hypothermia [13], and are not relevant to syndromic surveillance of febrile disease. Second, we removed all but the last of any sequence of temperatures logged <15 s apart (15.8 %, *n* = 14,206). Temperatures taken this quickly could only be repeated measurements of the same patient, and the last measurement would most likely be the temperature that was accepted by the clinician. Therefore, only the last temperature was retained for our analysis. After removing these measurements, 71,865 temperatures (80.0 %) remained for analysis. Note that this filtering does not affect the external generalizability of our results because they could be applied in real time to temperature surveillance deployed in any ED.

### Definition of fever

Following convention, common fever was defined as a body temperature ≥100.4 °F (≥38.0 °C). Hyperpyrexia (sometimes known as extreme hyperpyrexia) was defined as a body temperature ≥106.0 °F (≥41.1 °C) [14]. All temperatures were measured in degrees Fahrenheit. Celsius values appearing in this paper are rounded conversions of the Fahrenheit measurements.

### Disease outbreaks

We determined to compare the fever data with outbreaks of febrile disease occurring during the study period. Although a variety of febrile diseases are monitored internationally, weekly surveillance data was not readily available in Massachusetts for febrile diseases other than influenza. We therefore compared the prevalence of fever during periods of influenza activity with non-influenza periods. The collected data included two outbreaks of influenza: the autumn–winter wave of the H1N1 pandemic and a seasonal flu outbreak.

### Comparison with existing syndromic surveillance

Fever data from the ED were compared with existing syndromic surveillance for influenza. (If substantial outbreaks of other febrile diseases had occurred in Boston during the study period, comparisons would also have been made with any available surveillance data for these diseases.) The CDC reports data on the percent of outpatient visits for ILI at New England clinics participating in the Influenza-like Illness Surveillance Network (ILINet), including thresholds for periods of elevated influenza activity. Based on these thresholds, September 14, 2009–December 6, 2009 was the period of the autumn–winter wave of the 2009–2010 H1N1 pandemic in New England, and January 24, 2010–March 13, 2011 was the period of the 2010–2011 seasonal flu outbreak in New England [4]. We compared fever prevalence during these periods and during periods when influenza activity did not exceed the regional threshold (which are termed non-influenza periods for brevity).

In addition, fever data from the ED were compared qualitatively with weekly influenza reports from the Massachusetts Department of Public Health (MDPH) [15–17]. The MDPH reports provided data from the Automated Epidemiologic Geotemporal Integrated Surveillance System (AEGIS), including the percentage of total visits to EDs at 19 Massachusetts hospitals that were due to flu-like symptoms. The MDPH reports also provided weekly rates of ILI based on data from 46 (in 2009–2010) and 45 (in 2010–2011) hospitals, private physicians’ offices, and school health centers across Massachusetts [18]. Further, the MDPH reports provided weekly counts of influenza cases that were confirmed by laboratory testing (cultures and rapid tests) at the William A. Hinton State Laboratory Institute, providers’ offices, and laboratories across Massachusetts. It is worth noting that most cases of suspected influenza are not tested, and that the count of laboratory-confirmed cases is therefore much lower than the actual number of influenza cases in Massachusetts. It should also be noted that healthcare providers sometimes neglect to submit influenza reports to state surveillance systems. For example, 7 and 3 of the Massachusetts ILI reporting sources submitted reports for <16 weeks of the traditional flu reporting seasons in 2009–2010 and in 2010–2011, respectively [18], and it is likely that the AEGIS data were affected by missed reports from some EDs as well.

### Detection of aberrant fever rates

Originally, a prospective validation analysis was designed to estimate the requirements for successful detection of increases in the underlying fever rate. However, after several months of data had been collected, it became apparent that the fever rates in the data were substantially higher than those that had been assumed when designing the prospective analysis, and were also subject to fewer rapid changes. The prospective analysis was therefore abandoned, and attention shifted to comparing the fever rates observed in the ED data with the CDC and Massachusetts-level data sources that are discussed above.

In addition, an outbreak detection algorithm was applied to the data as a supplemental investigation (Additional file 1: Aberrant Event Detection Analysis).

### Statistical analysis

It was necessary to smooth the fever rates to make them visually interpretable. Smoothed estimates of fever rates were obtained via two methods: First, we computed the simple means of fever rates over the past week. Second, we applied exponential smoothing methods to the data. In practice, there are a variety of exponential smoothing methods, such as simple exponential smoothing and Holt’s linear method. We used a state space approach [19] as implemented in the R package forecast [20] to automatically select the exponential smoothing method and parameters that offered the lowest Akaike information criterion value for the fever data. In addition to the smoothing, we performed a simple analysis of the seasonality of fever rates, as discussed in the second supplemental appendix (Additional file 2: Seasonality). Although the results were not conclusive, we found no evidence of seasonality that was strong enough to warrant consideration in the analysis of fever rates.

Proportions were compared using the Chi-squared test and means were compared using the Mann–Whitney *U* test. Values of *p* < 0.05 were considered statistically significant and all tests were two-sided. The statistical analysis was performed in R (version 3.2.1; R Foundation for Statistical Computing, Vienna, Austria).

## Results

### Characteristics of body temperatures recorded in the ED

Between September 10, 2009 and August 29, 2011, 71,865 body temperatures were electronically recorded by the automatic data logging system and met the inclusion criteria (daily mean: 100.1 temperatures, daily SD: 35.3, daily range: 13–181). During the study period, there were 110,465 visits to the emergency department, 37 % of which resulted in admission (Table 1).

### Temperature distribution analysis

The mean body temperature was 98.1 °F (36.7 °C) with a standard deviation of 1.1 °F (0.6 °C). The median body temperature was 98.0 °F (36.7 °C) with an interquartile range of 97.4–98.7 °F (36.3–37.1 °C). These values are consistent with previous reports [21] (and it is worth noting that 98.6 °F [37.0 °C] is not the mean human body temperature, despite widespread belief [22]). Overall, 2073 fevers (body temperature ≥100.4 °F, ≥38.0 °C) were observed, constituting 2.6 % of the temperature recordings (daily mean: 2.9 %, SD: 2.1 %, range: 0.0–18.6 %). A mean of 1.0 fevers was measured per thermometer each day (SD: 0.9, range: 0.0–8.0).

Hyperpyrexia (body temperature ≥106.0 °F, ≥41.1 °C) generally constitutes a medical emergency. Because hyperpyrexia is exceptionally rare, and because patients with hyperpyrexia are likely to have their temperatures measured repeatedly, we considered all measurements of hyperpyrexia occurring within the same 12 h to be of the same patient. Other than repeated measurements within the same 12 h, all measurements in the hyperpyrexic range were at least 76 h apart (mean separation: 36.3 days). This analysis revealed 25 cases of hyperpyrexia, amounting to an incidence of 1 in 2875 temperature measurements recorded in the emergency department (34.8 per 100,000; 95 % CI: 22.5–51.4). This result from an adult ED is consistent with the higher rate of 1 in 1270 patient visits for hyperpyrexia found in a pediatric ED [14].

### Comparison with existing syndromic surveillance

**Fig. 1:**
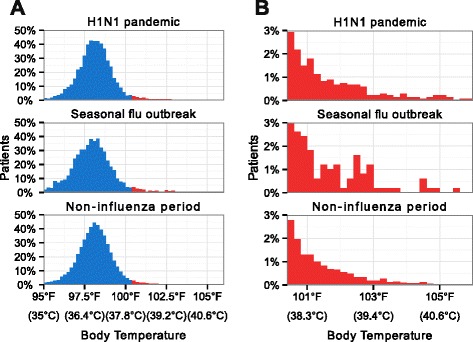
Temperatures collected during influenza epidemic periods and other periods. Panel (**a)** presents the distribution of all temperatures collected at a Boston emergency department during the autumn–winter wave of the 2009–2010 H1N1 pandemic in New England (September 14, 2009–December 6, 2010), 2010–2011 seasonal flu outbreak in New England (January 24, 2010–March 13, 2011), and periods without unusually elevated influenza activity (remaining dates between September 10, 2009 and August 29, 2011). Fevers are shown in red and non-fevers are shown in blue. For clarity, panel (**b)** presents the results for fevers only. Each period was defined based on data from the CDC’s Outpatient Influenza-like Illness Surveillance Network (ILINet)

Based on data from ILINet, September 14, 2009–December 6, 2009 was the period of the autumn–winter wave of the 2009–2010 H1N1 pandemic in New England, and January 24, 2010–March 13, 2011 was the period of the 2010–2011 seasonal flu outbreak in New England [4]. Fig. 1 presents the distribution of all temperatures (panel A) and fever temperatures (panel B) collected during these periods.

Although periods of influenza activity are likely to differ between Boston and greater New England, somewhat elevated fever activity was observed in the Boston ED temperature data during the periods of influenza outbreaks in New England. During the H1N1 pandemic period, the seasonal influenza outbreak, and the periods without elevated influenza activity, fevers accounted for 3.5 %, 4.2 %, and 2.8 % of temperatures, reflecting an elevated incidence of fever during both the H1N1 period and the seasonal influenza outbreak (H1N1 vs. non-influenza: *p <* 0.001, seasonal vs. non-influenza: *p <* 0.001). Further, the mean fever temperatures were 101.7 °F, 101.6 °F, and 101.4 °F, respectively (38.7 °C, 38.7 °C, and 38.6 °C, respectively), indicating a small but statistically significant increase in the severity of those fevers occurring during the H1N1 pandemic in New England (H1N1 mean vs. non-influenza mean: *p =* 0.01*,* seasonal mean vs. non-influenza mean: *p =* 0.10). Finally, daily means of 1.2, 2.2, and 0.9 fevers per thermometer were recorded during the H1N1 period, the seasonal influenza outbreak, and the periods without elevated influenza activity, showing a significant increase in the rate of fever measurements during the seasonal influenza outbreak (H1N1 vs. non-influenza: *p <* 0.17, seasonal vs. non-influenza: *p* < 0.001).

**Fig. 2:**
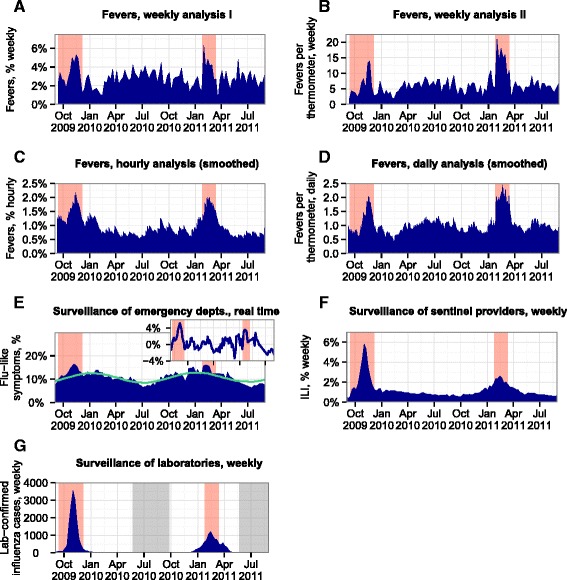
Fevers observed at the Boston-area emergency department, as compared with influenza surveillance in Massachusetts. **a** Fevers, weekly analysis I: weekly proportion of temperature measurements that were fevers. **b** Fevers, weekly analysis II: weekly number of fevers measured per thermometer. **c** Fevers, hourly analysis (smoothed): exponential smooth of the hourly proportion of temperature measurements that were fevers. **d** Fevers, daily analysis (smoothed): exponential smooth of the daily number of fevers measured per thermometer. **e** Surveillance of emergency departments, real time: proportion of patients with flu-like symptoms at 19 emergency departments in Massachusetts. The data are shown in blue, the seasonal trend is shown in green, and the data with the seasonal trend removed are shown in the inset. **f** Surveillance of sentinel providers, weekly: proportion of visits for influenza-like illness at more than 40 hospitals, private physicians’ offices, and other providers across Massachusetts that participated in the sentinel surveillance program. **g** Surveillance of laboratories, weekly: laboratory-confirmed cases of influenza. Periods for which data were not reported are shaded gray. In all plots, the orange bands display the CDC-defined periods of elevated influenza activity during the H1N1 (swine flu) pandemic (at left) and a seasonal flu outbreak (at right)

Figure 2 compares temperature data collected from the ED with influenza surveillance reports in Massachusetts. Since the ED data were obtained as exact temperatures and measurement times, they can be used to calculate many different forms of fever rates. Panels A and B present two such forms of fever rates: panel A shows the weekly proportion of all temperatures measurements in the ED that were fevers, while panel B shows the weekly number of fevers that were measured per thermometer. Peaks in the fever rates are evident during the H1N1 pandemic period and the seasonal influenza period, which are shown as the shaded orange bands on the graphs.

The fever data in panels A and B were summarized on a weekly basis to make them more directly comparable with the influenza surveillance data in the figure, which were also compiled and reported weekly. However, the weekly summaries of the fever data discard valuable information that is available on the exact measurement times and days. To show the benefits of this information, we have displayed exponential smooths of fever rates at shorter intervals in panels C and D. Panel C displays an exponential smooth of the hourly proportion of temperature measurements that were fevers, while panel D displays an exponential smooth of the daily number of fevers measured per thermometer. The Akaike information criterion was used to select the parameters and methods of exponential smoothing for the hourly proportion and daily rate data, which were simple exponential smoothing with additive errors and multiplicative errors, respectively. As compared with the weekly data in panels A and B, the hourly and daily data in panels C and D show more sustained peaks in the fever rate during the periods of elevated influenza activity, as well as fewer strong peaks outside of the epidemic periods.

Panel E of Fig. 2 shows data from the AEGIS flu system, which collected real-time data on the percentages of total visits to 19 EDs that were due to flu-like symptoms. The AEGIS data exhibit strong seasonality and were published with a seasonal baseline for adjustment (shown in green). In the panel inset, this baseline has been removed from the data. In both the unadjusted and adjusted AEGIS data, there is a peak in flu-like disease during the H1N1 period, but it is difficult to distinguish the rates of flu-like symptoms during the seasonal influenza outbreak from those outside of the epidemic periods. Overall, thermometer data collected from a single ED appeared to provide clearer indications of elevated influenza activity in 2009–2011 than did the rates of flu-like disease that were collected from 19 EDs.

Panel F of Fig. 2 shows weekly data on the percentage of visits due to ILI, as reported by more than 40 hospitals, private physicians’ offices, and other providers across Massachusetts. These data show unambiguous peaks in the ILI rates during both the H1N1 pandemic period and the seasonal influenza period. Panel G shows state-level counts of laboratory-confirmed influenza cases, which also peak unambiguously during the periods of elevated influenza activity. In summary, these data provide clearer indications of influenza activity than do the fever rates from a single ED or the AEGIS rates of flu-like symptoms.

### Detection of aberrant fever rates

Judged based on the appearance of the fever data alone, it was not clear whether fever rates from a single ED would be sufficient to detect significant increases in febrile disease, or whether the detected increases would correspond with the periods of elevated influenza activity during the period of the study. To address these points, we performed a supplemental analysis of the fever data using a Bayesian outbreak detection algorithm (Additional file 1: Aberrant Event Detection Analysis) [23, 24]. In brief, the analysis successfully detected significant increases in fever rates, which corresponded with both the autumn–winter wave of the H1N1 pandemic and the seasonal influenza outbreak. However, temperature data from additional settings and years would be needed to establish fever rates as a means of rapidly detecting outbreaks of influenza or other specific diseases.

## Discussion

Here, we have described a method of automated temperature collection and the successful application of this method to monitor fever rates in an active ED over the course of 2 years. The collection of temperature measurements during the H1N1 outbreak of 2009–2010 provided a unique opportunity, and notable increases in fever rates were detected both during the regional outbreak of this pandemic and during the period of widespread seasonal influenza transmission in 2010–2011. In qualitative comparisons of the fever rates with influenza surveillance programs in Massachusetts, the fever rates appeared to provide a stronger signal than some reported markers of influenza (flu-like symptoms), but provided a substantially weaker signal than others (ILI rates and counts of laboratory-confirmed influenza cases). On the other hand, the clearer signals from ILI rates and laboratory-confirmed cases came at the cost of requiring manual reports from a large number of providers across Massachusetts, which can be burdensome to healthcare professionals [18], is inapplicable to community-level disease surveillance, and relies upon the existence of a well-developed medical infrastructure.

If the system described in this study were expanded, a network of wireless-enabled thermometers could automatically send temperatures, times, and locations to a central server, providing essentially real-time data without requiring any action from clinicians beyond normal patient care. Because body temperature is simple to measure, temperature-based surveillance might be suitable for schools, workplaces, transportation hubs, and other non-clinical and non-expert settings, in which many other means of real-time syndromic surveillance would be infeasible. Because access to cellular and other wireless networks is widespread, the system might also be suitable for regions where low medical infrastructure has been an obstacle to timely disease surveillance. Temperature is an indicator for many diseases, including contagious diseases that present pandemic concerns. Further, temperature can be measured in ways that present little risk of disease transmission from the patient. Yet, extending this initial study’s results to active detection and characterization of outbreaks remains a topic for future research.

Despite the unusually large sample size of collected temperatures (*n* = 71,865), the present study was limited by its single-institution design, and it is difficult to say how the results would generalize to different communities. Data were collected from patients who registered via triage in the ED. The sickest patients often bypass triage, and therefore their temperatures were not necessarily recorded in this study. Additionally, data on laboratory tests for febrile diseases at the ED were not available to confirm the fever rate results. Further, our analysis only considered two outbreaks of febrile disease, both of which were of influenza, importantly limiting our ability to assess the system’s performance.

## Conclusions

We have presented a novel method of automated temperature collection, demonstrated that it can be used in the setting of an active emergency department, and shown that peaks in the resulting fever rates correspond with elevated regional rates of influenza. The temperature data showed promise as a potential surveillance tool that could complement current systems for monitoring febrile disease in the community, including systems that aim to detect outbreaks of febrile disease quickly. Febrile disease is important to monitor because this category includes seasonal influenza—which leads to yearly increases in respiratory-related mortality [25], morbidity [26], medical costs [27], and workplace absenteeism [28]—as well as emerging and worsening health threats, such as dengue fever [12, 29, 30], avian influenza, SARS [31], and Ebola. Nonetheless, additional studies are needed to validate real-time surveillance of body temperature and assess the generalizability of the results in this paper to other emergency departments and epidemic periods. It may be particularly fruitful to evaluate the system in non-expert settings, such as workplaces and transportation hubs, and in regions where low medical infrastructure is an obstacle to traditional disease surveillance.

## Additional files

Additional file 1: Aberrant event detection analysis. (PDF 467 kb) Additional file 2: Seasonality. (PDF 111  kb)
