# Supplementary material for: Human temperatures for syndromic surveillance in the emergency department: data from the autumn wave of the 2009 swine flu (H1N1) pandemic and a seasonal influenza outbreak
Source: BMC Emerg Med. 2016 Mar 9;16:16. doi: 10.1186/s12873-016-0080-7 (PMC4784270; doi:10.1186/s12873-016-0080-7)
Supplement: Additional file 1: — Aberrant event detection analysis. (PDF 467 kb) [file 12873_2016_80_MOESM1_ESM.pdf]

Human temperatures for syndromic surveillance in the emergency department: data from the autumn wave of the 2009 swine flu (H1N1) pandemic and a seasonal influenza outbreak

Bordonaro SF, McGillicuddy DC, Pompei F, Burmistrov B, Harding C, Sanchez LD

Additional File 1: Aberrant Event Detection Analysis

Introduction

Judged based on the appearance of the data alone, it was not clear whether fever rates from a single ED would be sufficient to detect significant increases in febrile disease, or whether the detected increases would correspond with the periods of elevated influenza activity during the period of the study. To address these points, we retrospectively applied Manitz and Hohle's Bayesian outbreak detection algorithm (BODA) [1, 2]. This algorithm generates alarms signalling that the underlying process is out-of-control relative to the recent past. Alarms generated by the algorithm were compared with two outbreaks that occurred during the study period—the autumn-winter wave of the 2009 swine flu (H1N1) pandemic and the seasonal influenza outbreak of 2011.

Methods

Technical details of BODA

Conceptually, BODA is a Bayesian extension of the well-known Farrington algorithm of outbreak detection [3]. In essence, BODA works by learning the expected distribution of fevers in a non-epidemic period, and then checks whether the current rate of fevers is statistically consistent (in control) or inconsistent (out of control) with this expected distribution, while using Bayesian updating to incorporate information on the fever rates during previous days and account for both estimation and prediction uncertainty.

BODA was selected for several reasons: First, because it allowed the false alert criterion to be specified in the same manner as in the design of our original validation analyses. Second, because it accommodates overdispersion, which was observed in the data. Third, because it allowed us to incorporate the number of in-use thermometers within the algorithm. Fourth, because it is widely available in the R surveillance package [4]. Finally, because it has few adjustable parameters in the surveillance package, which decreases experimenter degrees of freedom. We viewed it as especially important to reduce experimenter degrees of freedom given the limited number of outbreaks that were analyzed in our study (n = 2). The parameters were chosen *a priori*—they were not tuned.

The details of each BODA implementation were as follows: A negative binomial generalized additive model was used to predict the count of expected fevers and learn non-epidemic (in-control) parameters for use by the algorithm. These non-epidemic parameters were learned from the last 12 weeks of the study. Although it would be more common to learn parameters from the first weeks of data, this was not possible because the autumn wave of the H1N1 epidemic coincided with that period. The number of thermometers used during each time segment (week or day) was included in BODA as a covariate. The probability of a false alert was set at ≤3 per year for the main analysis and at 1–5 per year for secondary analyses.

True alerts and false alerts

True alerts were defined as alerts triggered during weeks for which ILI activity in New England exceeds the CDC’s threshold [5]. False alerts were defined as alerts triggered during any other period. The algorithm’s parameters were set to allow ≤3 false alarms per year.

Since fever rates may be elevated by diseases other than influenza, the definitions of true and false alerts are practical approximations, rather than gold standard ideals. However, it was

necessary to develop some criteria for true and false alerts, even if imperfect, because these definitions are required to evaluate the performance of an aberrant event detection analysis.

Robustness and timeliness of outbreak detection

Two analyses were performed to examine the robustness and timeliness of outbreak detection using real-time fever data. To examine robustness, we re-ran the outbreak detection analysis with various acceptable rates of false alerts, ranging from ≤1 to ≤5 per year. We then checked whether outbreaks continued to be detected across the range of allowed false alert rates. To examine timeliness, we ran the outbreak detection analysis twice. First, we ran it on fever data that had been binned into single week intervals, simulating the case of having data available on a weekly basis, which is currently the most common situation in US biosurveillance of influenza and some other diseases. Second, we ran the BODA on fever data that had been binned into 1-day intervals as a proxy for real-time availability. We then compared the earliest dates at which increased fever rates were detected during the periods of the influenza outbreaks, using the weekly and daily data with various false alert criteria.

Results

Additional Figure 1 presents the results of applying a Bayesian outbreak detection algorithm (BODA) [1] to the number of fevers measured in the ED per thermometer. The light blue and dark blue areas show the weekly and daily rates of fevers, respectively. For example, data from the first week of August 2011 show that around 5 fevers were measured per thermometer. The outbreak detection algorithm produces an epidemic threshold (red lines) that is used to judge whether an outbreak is detected. When the observed fever rates exceed this threshold, an alarm is declared (red triangles). Using fever rates from the Boston ED, the algorithm successfully detected both the autumn–winter wave of the 2009–2010 swine flu pandemic and the seasonal flu

epidemic of 2011. Outbreak detection was successful for both the weekly data and the daily data, even though the daily data appear quite noisy to the eye.

False alarms are an important concern in outbreak detection, since they lead to wasted effort and potential "boy who cried wolf" situations. Here, we defined a false alarm as any alarm issued outside of the epidemic periods shown in the figure (see the Methods section for more details). A key advantage of the Bayesian outbreak detection algorithm is that it allows the expected number of false alarms to be established prospectively. In the figure, we arbitrarily employed the criterion that an average of ≤3 false alarms should be issued per year. Both the weekly and daily analyses satisfied this criterion (weekly, 0 false alarms; daily, 2 apparent false alarms in 2 years). In additional analyses, we employed false alarm criteria ranging from ≤1 to ≤5 per year. While the weekly outbreak detection analysis showed diminishing ability to detect the outbreaks for low false alarm criteria, the daily analysis detected both outbreaks for all of the false alarm criteria, suggesting that daily fever data provided robust evidence of these outbreaks (Additional Table 1).

To examine the timeliness that could be offered by using real-time data instead of weekly data, we compared the dates at which the algorithm was able to detect periods of elevated febrile activity based on daily and weekly rates of fever. As compared with the weekly data, the daily data provided 18 days of advanced notice for the H1N1 pandemic and 5 days of advanced notice for the seasonal flu outbreak with ≤3 false alarms per year. These indications are promising, especially because weekly CDC surveillance on influenza-like illness and laboratory-confirmed influenza is usually subject to several additional days of delay in practice, as the data are compiled. However, our analysis was importantly limited by the fact that our analysis only investigated two epidemic periods, both of which were of influenza.

Summary

In brief, the analysis successfully detected significant increases in fever rates, which corresponded with both the autumn–winter wave of the H1N1 pandemic and the seasonal influenza outbreak. The analysis suggests that temperature data could have potential as a surveillance tool for febrile illness, especially if it were employed in conjunction with other syndromic indicators. However, temperature data from additional settings and years would be needed to establish fever rates as a means of rapidly detecting outbreaks of influenza or other specific diseases.

References

1. Manitz J, Höhle M: **Bayesian outbreak detection algorithm for monitoring reported cases of campylobacteriosis in Germany**. *Biometrical J* 2013, **55**:509–526.

2. Maëlle S, Dirk S, Höhle M: **Monitoring count time series in R: aberration detection in public health surveillance**. *arXiv* 2014:1411.1292.

3. Farrington CP, Andrews NJ, Beale AD, Catchpole MA: **A statistical algorithm for the early detection of outbreaks of infectious disease**. *J R Stat Soc Ser A* 1996, **159**:547–563.

4. Höhle M, Meyer S, Paul M: **surveillance: temporal and spatio-temporal modeling and monitoring of epidemic phenomena. R package.** 2015.

5. **FluView** [http://www.cdc.gov/flu/weekly/]

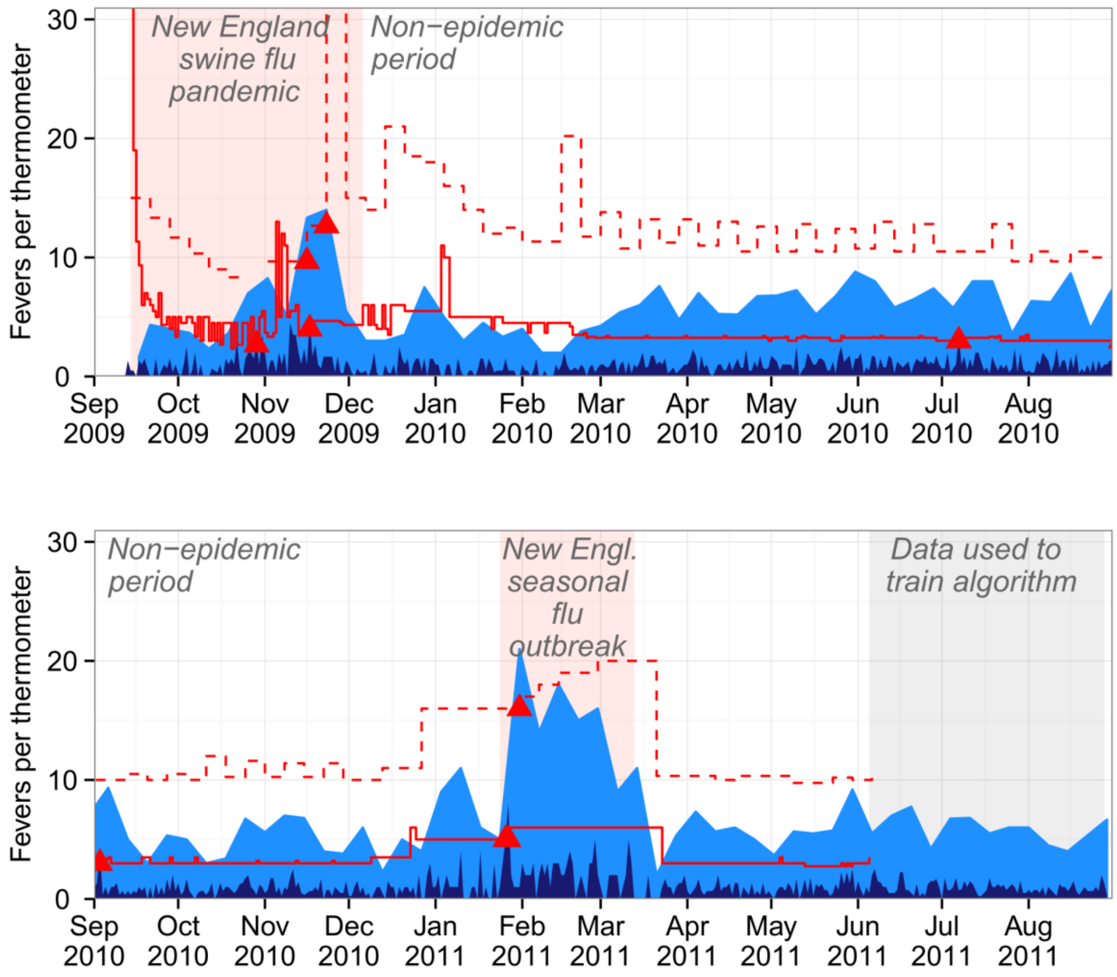

Additional Figure 1. An outbreak detection algorithm applied to rates of fever at the Boston-area emergency department. A Bayesian outbreak detection algorithm was retrospectively applied to weekly (light blue) and daily (dark blue) fever rates per thermometer. When the observed fever rates crossed the epidemic threshold generated by the algorithm (red lines), an alarm was declared (red triangles). Both of the known outbreaks of influenza (regions shaded red) were detected using the data from this emergency department. The daily data provided earlier alarms, and both the weekly and daily data satisfied the criterion of ≤3 false alarms per year.

Additional Table 1. Applying the Bayesian outbreak detection algorithm to daily and weekly fever rate data, with various false alarm criteria. Applying the detection algorithm daily detected both outbreaks in all cases. Further, the daily analysis generally provided advanced notice of outbreaks in comparison with the weekly analysis, or identified outbreaks that were undetected by the weekly analysis.

Outbreak detection analysis		***Results***					
Data type	*Maximum allowed false alarms per year**	*Observed false alarms per year***	*Outbreaks detected*	*Date swine flu pandemic detected*	*Date seasonal flu detected*	*Advanced notice of swine flu† (days)*	*Advanced notice of seasonal flu† (days)*
Weekly data	5	0.5	2/2	11-16-2009	1-31-2011	-	-
	4	0	2/2	11-16-2009	1-31-2011	-	-
	3	0	2/2	11-16-2009	1-31-2011	-	-
	2	0	1/2	11-16-2009	Undetected	-	-
	1	0	1/2	11-16-2009	Undetected	-	-
Daily data	5	1.0	2/2	10-29-2009	1-26-2011	18	5
	4	1.0	2/2	10-29-2009	1-26-2011	18	5
	3	1.0	2/2	10-29-2009	1-26-2011	18	5
	2	1.0	2/2	11-17-2009	1-27-2011	-1	Inf.
	1	0.5	2/2	11-17-2009	1-27-2011	-1	Inf.

* Criteria for acceptable rate of false alarms, as specified in the Bayesian outbreak detection algorithm.

** Observed rate of false alarms, including every alarm triggered during a period other than the CDC-defined swine flu pandemic and seasonal flu outbreak periods in New England.

† Advanced notice was calculated as the number of days of advanced warning provided by the analysis of daily data, as compared with the corresponding analysis of weekly data.

Inf., infinite because the outbreak was not detected in the weekly analysis.
