# Supplementary material for: Human temperatures for syndromic surveillance in the emergency department: data from the autumn wave of the 2009 swine flu (H1N1) pandemic and a seasonal influenza outbreak
Source: BMC Emerg Med. 2016 Mar 9;16:16. doi: 10.1186/s12873-016-0080-7 (PMC4784270; doi:10.1186/s12873-016-0080-7)
Supplement: Additional file 2: — Seasonality. (PDF 111 kb) [file 12873_2016_80_MOESM2_ESM.pdf]

Human temperatures for syndromic surveillance in the emergency department: data from the autumn wave of the 2009 swine flu (H1N1) pandemic and a seasonal influenza outbreak

Bordonaro SF, McGillicuddy DC, Pompei F, Burmistrov B, Harding C, Sanchez LD

Additional File 2: Seasonality

A simple analysis of the fever rate data was performed to look for evidence of seasonality, which is a common feature in disease surveillance. For the analysis, we fit the following negative binomial model to the data.

$$\log(\text{Fevers}) \sim \alpha + \beta' \text{ Month}$$

A negative binomial model was chosen to accommodate overdispersion in the fever rates, and the number of thermometers in use was included as an offset. Before fitting the model, we excluded all days in the New England H1N1 and seasonal flu periods that are seen in Figure 2 of the main article file.

Additional Figure 1 shows the relative rates that we found, with January as the reference category. There was no evidence of seasonally greater fever rates during winter, which is the most common seasonal pattern for influenza. Instead, the fever rates were lower than typical during January and February and were roughly similar through the rest of the year. Lower rates in January and February could simply reflect the usual heterogeneities and external events that are encountered in real-word surveillance data, such as changing practice patterns.

Although the results do not exclude the possibility that there is some seasonality in the data, we found no evidence of seasonality that was strong enough to require consideration in the analysis of fever rates. There was no evidence that the excess fever rates during influenza periods are explained by seasonal patterns.

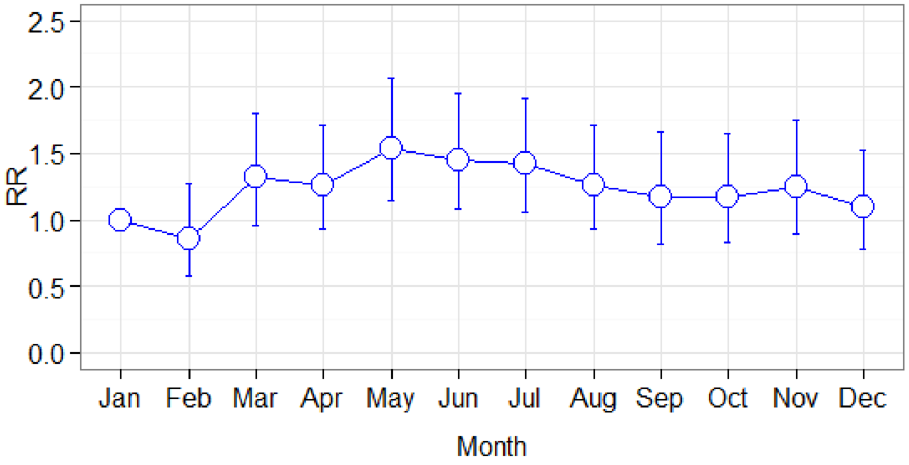

Additional Figure 1. Relative risks (RRs) of fever by month of the year, with January as the reference category. Confidence intervals are 95%.
